# Biomarkers of Inflammaging and Cellular Senescence in Musculoskeletal (MSK) Diseases: The Knowns and the Unknowns

**DOI:** 10.3390/biomedicines14071486

**Published:** 2026-06-30

**Authors:** Payal Ganguly

**Affiliations:** 1 Leeds Institute of Rheumatic and Musculoskeletal Medicine, University of Leeds, Leeds LS7 4SA, UK; p.ganguly@leeds.ac.uk; 2 Leeds Biomedical Research Centre, Leeds Teaching Hospitals NHS Trust, Leeds LS7 4SA, UK

**Keywords:** inflammaging, senescence, biomarkers, immunosenescence, bone aging, musculoskeletal (MSK), healthy aging, longevity, anti-aging

## Abstract

Advancing age, while a natural trajectory, often leads to several age-related diseases (ARDs) and impacts the quality of life (QOL) of the elderly. The World Health Organization (WHO) predicts that the incidence of ARDs is only going to increase over the next couple of decades. Musculoskeletal (MSK) diseases associated with advancing age are a major global health burden and are closely associated with cellular senescence, inflammaging and immunosenescence. To target these, a clear pathway with well-defined biomarkers that can then be translated into clinical applications is needed. Clearly defined biomarkers will bring us one step closer to dissecting age-related MSK changes, tracking these changes with advancing age, predicting these MSK ARDs and thus providing a platform towards healthy aging, disease-free life in the elderly and longevity. This review outlines our current knowledge in the field, discusses the current knowns and unknowns, provides an overview of the anti-aging strategies, and finally encourages its readers to consider approaches to help converge biomarkers of aging for clinical translation using next-generation technologies.

## 1. Introduction

Globally, advancing age is increasingly associated with several age-related diseases (ARDs) including neurodegenerative diseases [1,2], cardiovascular diseases [3,4] and musculoskeletal (MSK) diseases [5,6]. MSK diseases account for among the largest global burden of diseases, impact mobility, reduce social interaction and quality of life (QOL), and negatively affect patient mental health [7,8]. For several MSK ARDs, including osteoarthritis (OA), osteoporosis (OP), rheumatoid arthritis (RA) and others, both senescence and inflammaging have been reported to be involved in their progression [9,10,11,12]. While advancing age is a completely natural phenomenon and there are several people over the age of 60 that are fit, strong and healthy, the transition from healthy aging to frailty and ARDs is a puzzle that we are yet to fully comprehend. This is where the difference between chronological age (one’s actual numerical age) and biological age (level of molecular damage occurred) begins to play an important role [13].

Research in the last couple of decades has made significant advancements in this field with the ‘hallmarks of aging’ pioneering the different features and theories that contribute to age-related changes at the cellular level. These broadly include primary hallmarks that cause damage (genomic instability, telomere attrition, epigenetic alterations and loss of proteostasis), antagonistic hallmarks that are the responses to this damage (dysregulated nutrient sensing, cellular senescence and mitochondrial dysfunction), and integrative hallmarks that are culprits of the phenotype (stem cell exhaustion and altered intercellular communication) [14]. A decade later, the authors have updated these under the title of ‘an expanding universe’ with the addition of disabled macro-autophagy, dysbiosis and chronic inflammation (or inflammaging) [15]. Among these, cellular senescence and inflammaging have been closely linked with several ARDs [16,17,18].

**Figure 1:**
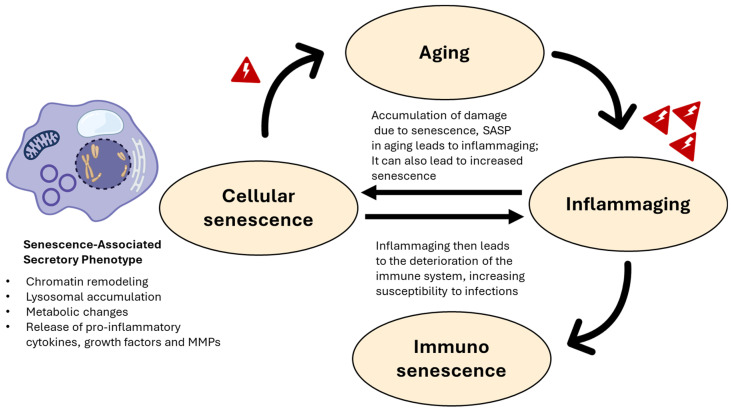
Links between aging, inflammaging, cellular senescence and immunosenescence.

Inflammaging is defined as chronic low-grade inflammation that accumulates with advancing age and is increasingly being associated with several ARDs [19,20]. Cellular senescence is the process in which cells lose their ability to proliferate irreversibly [21,22]. While the process is needed for performing different functions, accumulation of senescent cells over time leads to the secretion of senescence-associated secretory phenotype (SASP) [23,24]. Senescence and SASP have now been known, defined and quantified in the last couple of decades. Both senescence and inflammaging are known to contribute towards several ARDs, with inflammaging being a more recent concept than the former. They appear bi-directional in their cause and effects with respect to ARDs, resulting in immunosenescence [18], which is the age-related decline in the functioning of the immune system (Figure 1), but senescence is currently more quantifiable due to specific markers and known senescent cellular features.

With the increasing interest in the field, several scientists and research groups have contributed to the growing body of evidence towards cellular senescence and inflammaging in MSK. Existing evidence indicates that senescence might be the key to tracking diseases (and thus targeting senescence for treatment), healthy aging and longevity; however, this knowledge is yet to be translated to the ‘bedside’ for clinical applications. This review aims to outline and simplify our current understanding of the complex links between aging, senescence and inflammaging in MSK diseases, classify and list the biomarkers of aging, and discuss the knowns and the unknowns of the field. Finally, this review underscores the challenges, conclusions and future directions, to enable clinical translation, targeted biomedical therapeutics to envision healthy MSK aging and to bring longevity within reach.

## 2. Biomarkers of Inflammaging and Cellular Senescence

### 2.1. Definition of Biomarkers and Their Classification

**Figure 2:**
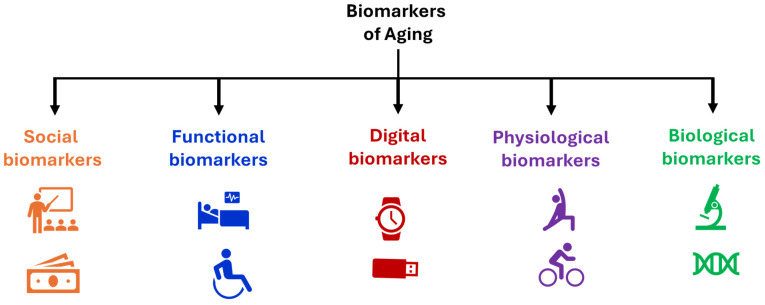
Simple classification of biomarkers of aging.

Biomarkers of aging broadly may be classified as social, functional, physiological and biological [25,26,27]. Social biomarkers include socio-economic factors including educational and economic background, which comes under social aspects and deserves a separate manuscript altogether. Functional biomarkers include ARDs, system functions and sensory functions; physiological biomarkers include physical performance-based observations (grip strength, hair loss, wrinkles, organ level changes); and biological biomarkers dissect cell and molecular pathways, inter- and intracellular networks and the hallmarks of aging. A recent review discussed several parameters (including cholesterol, blood pressure, circadian rhythm and others) that could be monitored digitally via a smart watch or a smart phone app to assess age-related changes, referring to these as the digital biomarkers of aging (Figure 2) [13].

While an attempt has been made to classify these biomarkers, there is bound to be some overlap between them. For example, several functions like heart rate and blood pressure are categorized as physiological markers. However, with the current smart watches and applications focused on tracking and monitoring them online, they are also categorized under digital biomarkers. Scientists and researchers in the field must acknowledge the dynamism of this ever-changing field while defining and classifying these biomarkers.

The aging biomarker consortium defines aging biomarkers as a combination of biological parameters to assess age-related changes, to track the physiological aging process and to predict the transition to a pathological state. The researchers also proposed that these biomarkers ‘should qualify for being specific, systemic, and clinically relevant’ [28]. Our focus will be biological biomarkers of aging, senescence, immunosenescence and inflammaging within MSK, all of which are intricately linked as shown in the figure above.

While there is an increasing interest in how these are mechanistically linked, research investigations to establish the quantification of senescence and inflammaging are limited (Table 1). This implies that even though the potential clinical implication of dissecting these phenomena is high, the path to clinical translation is yet to be navigated.

### 2.2. Senescence and Inflammaging Biomarkers

One of the challenges we face in the clinical translation of targeting senescence and inflammaging is the limited or lack of biomarkers that have been established, evaluated and standardized. This is due to several factors, one of them being that different organs age differently and vary in the signatures in which they age, express inflammation and senesce, i.e., heterogeneity in aging patterns. Thus, the blood- and serum-based markers of interleukin-6 (IL-6), interleukin -1 (IL-1), tumor necrosis factor alpha (TNFα) and C-reactive protein (CRP), which are established inflammation markers that can be collected as a standard sample from patients, are also considered as markers of inflammaging [38,39]. For cellular senescence, measurement of senescent cells using the senescence-associated beta-galactosidase (SA-β-gal) assay, markers including p53, p16 and p21, anomalies in cell morphology, and measurement of live and dead cells are commonly used to distinguish senescent cells [40,41].

p16, p21 and p53 are key markers known to be involved in the cell cycle and DNA damage response (DDR), acting as cell cycle checkpoints [42]. p21 is known to have dual roles in the cell cycle, with higher levels inhibiting cyclin D1-CDK4 complex leading to cell cycle arrest, and lower levels facilitating cell proliferation [43]. Evidence suggests that p21 can influence cell mitosis fate and division under radiation, indicating its significance as a specific marker to evaluate DNA damage and possibly cell fate [44]. Senescent cells have been shown to have upregulated levels of cyclin-dependent kinase inhibitors (CKIs) like p16 and p21, indicated by upregulated mRNA levels of p16^INK4a^ and p21 in elderly humans compared to young women [45]. p53, followed by p16, has been indicated to play a stronger role in senescence response to telomere dysfunction than p21 [46]. Accumulation of p16^INK4a^-positive cells was demonstrated in an in vitro experiment with mature chondrocytes in an OA progression model [47]. In transgenic mice, p21 overexpression in senescent cells corroborated tissue fibrosis, low levels of skeletal muscle mass and reductions in physical function [48]. Bone marrow mesenchymal stromal/stem cells (BM-MSCs) without p53 were reported to alter bone remodeling and impact cancer-associated bone modeling via negative regulation of osteoprotegerin (OPG) [49].

Molecular biomarkers of aging include telomere length and telomerase activity; transcripts including the levels of genes like *Klotho* and *Sirtuins*; and genetic variations like single nucleotide polymorphism. Cellular biomarkers of aging include senescence and SASP, reactive oxygen species (ROS) and oxidative stress in mitochondria, and reduction in numbers and functionality of stem and immune cells [26,50,51]. *Klotho* is a widely known and accepted anti-aging gene with key functions in bone metabolism and cellular senescence. Expressed in all cells of the bone lineage, from BM-MSCs to osteoblasts and osteocytes, *Klotho* is known to regulate bone formation [52]. Loss of *Klotho* has been reported to be associated with alteration of bone architecture and its turnover [36]. *SIRTs* (*Sirtuins*) including Srit 1–7 are a family of nicotinamide adenine dinucleotide (NAD+)-dependent protein deacetylases, responsible for a wide variety of cellular processes including cell metabolism, development and cellular senescence [53]. *SIRT-1* activation has revealed a protective effect in OA by enhancing the trabecular and subchondral bone and by inhibiting apoptosis of the chondrocytes [54].

For the MSK system specifically, the markers may be broadly classified between muscles and bone. Age-related muscle loss or sarcopenia is a widely investigated phenomenon and its effects have been reported to be potentially combated by the right diet, nutrition and exercises [55,56]. As a tissue, muscle is accessible and thus a large number of studies have investigated sarcopenia. However, muscles are held together by the skeletal framework of bones, and age-related bone loss is known to contribute to several MSK ARDs [57,58,59]. Research from our group has highlighted the role of BM lesions and targeting subchondral bone in MSK diseases [60,61], stem cell exhaustion in the BM observed by a decline in number in BM MSCs [62], DNA damage response (DDR) pathways involved [63], and the potential of biomaterials and biologics to combat senescence [64,65,66]. Another disease closely associated with MSK aging is type 2 diabetes (T2D). T2D is increasingly being acknowledged as a disease of accelerated aging, specifically linked with OP as they can co-exist [67,68]. T2D is driven by hyperglycemia and insulin resistance causing oxidative stress, inflammation, and advanced glycation end-product (AGE) accumulation. These impair differentiation capacity of osteoblasts, enhance osteoclast activity and promote cellular senescence in BM progenitors. Thus, this disrupted bone remodeling along with reduced bone quality and stem cell exhaustion links T2D with accelerated OP and skeletal aging [69].

Last year, an expert consensus statement on the biomarkers of aging was published, specifically for use in interventional studies, done by the Delphi methods in three different phases. The expert panel identified 14 biomarkers across three different rounds and these included the following: insulin-like growth factor 1 (IGF-1), growth differentiation factor 15 (GDF-15), high-sensitivity CRP, IL-6, DNA methylation/epigenetic clocks (biological markers), muscle mass, muscle strength, time-up-and-go, hand grip strength, gait speed, standing balance test, frailty index (functional markers), cognitive health (Montreal cognitive assessment) and blood pressure (physiological markers) [70]. This expert consensus statement will undoubtedly enable the scientific community to measure age-related changes and define parameters for standardized tests. Similar consensus statements re-iterating on factors to measure inflammaging and senescence will also be extremely useful in research. While some overlap is expected, defining the latter will help narrow down molecular pathways and targets that are involved in the transition from aging to ARDs [71].

### 2.3. Emerging Biomarkers

Another set of biomarkers that is currently being investigated and will evidently form a major part of the field is the set of next-generation aging biomarkers. These include extracellular vesicles (EVs), DNA methylation, epigenetic clocks and gut microbiome potentially for OP and sarcopenia [72]. With respect to bone, bone turnover markers like procollagen type I N-propeptide (PINP) and C-terminal telopeptide (CTX) reflect dynamic remodeling processes and are increasingly used in osteoporosis risk prediction and monitoring [73]. Advances in omics have introduced epigenetic clocks, based on DNA methylation, which quantify biological aging and show promise for detecting accelerated aging in MSK tissues [74]. Additionally, digital biomarkers derived from wearable sensors, such as gait speed and variability, provide real-world measures of functional decline and predict outcomes like frailty and falls [13]. Overall, the field is shifting toward multimodal biomarker panels integrating molecular, structural, and functional data.

## 3. The Knowns and the Unknowns

### 3.1. The Knowns

Over the last couple of decades, we have been able to determine the definitions of processes that have led to deterioration of health, reduced QOL, caused cellular and molecular damage and eventually resulted in several ARDs. While aging sets the stage, chronic low-grade inflammation or inflammaging negatively impacts the immune system [75,76]. Damage at the cellular level accumulates to make cells senescent and these senescent cells produce inflammatory SASP that leads to further damage and MSK ARDs [77,78,79]. All of these together cause a decline in the immune strength and that leads to immunosenescence. As discussed in the sections above, cellular senescence is better characterized with specified identifiable changes including the blue stain of the SA-β-Gal assay, cell morphology, and the cell cycle checkpoint markers of p16, p21 and p23. Thus, we know some of the terms, but we are yet to create a pathway to translate this from bench to bedside, especially considering that the current defined biomarkers of inflammaging are essentially the same markers that are used to identify inflammation (IL-6, TNF-α and CRP).

With respect to the MSK system, we know that cellular senescence and inflammaging drive MSK aging, contributing to diseases like OA, OP and RA [12,80,81,82,83]. This leads to increase in SASP which in turn increases the risk of inflammaging, resulting in tissue degeneration and compromised ability of repair and healing. With advancing age, the immune system weakens, and senescence and inflammaging further impair the immune system, now referred to as immunosenescence in the MSK system [84,85]. This leads to a vicious cycle that exacerbates MSK aging and the associated ARDs.

We also know that there is an increased interest in strategies and interventions that may combat this damage at the cellular level. Pharmacological approaches, use of biologics like platelet-rich plasma (PRP) and the use of diet, nutrition and exercise are all being explored simultaneously by different researchers globally [12,86,87,88]. However, the pathways followed by each of these interventions to reduce inflammaging and cellular senescence remain to be investigated.

A recent report discusses the ‘hallmarks of inflammaging’ as a result of an inflammaging workshop held in September 2025 to explore inflammaging, its mechanisms and intervention strategies. The authors suggest that inflammaging that leads to immune-aging, or immunosenescence, is now increasingly becoming measurable with our increased knowledge in the field, especially in the immune cells. Thus, this is an angle that must be dissected further to provide clarity to these biomechanistic links between advancing age and inflammaging, senescence and immune-aging [89].

### 3.2. The Unknowns

The mechanistic links and markers that connect aging to inflammaging, senescence and immunosenescence are currently limited. In spite of our knowledge about some of the interleukins as markers, the exact pathways and the precise causes are yet to be delineated. As members of the scientific community, it is essential to consider several factors that are currently unknown or yet to be established in the field. These include the multi-aging nature of our body, where all the organs age at their own rate. This also implies that using a single biomarker will likely be insufficient and we will need a ‘panel’ of markers for indicating inflammaging. This directly feeds into the challenge of biological variability of any of these biomarkers, especially circulating cytokines, suggesting that single tests will be insufficient to predict inflammaging accurately.

Next, there are limited cell and molecular biomarkers that are established and the ones that are established need further validation. To accurately measure senescence burden, inflammaging intensity, or immunosenescence activity in MSK tissues, no validated or disease-specific biomarkers currently exist. Inflammatory composition of SASP (IL-6, CRP, TNFα) varies across diseases and tissues, creating another barrier for clinical translation. Specifically for the BM, which is the core of the MSK system, our understanding of the interactions between the marrow niche, stem cells, and immune cells is continuously evolving. Thus, to narrow down ways in which age-related changes in the BM are affected by the bi-directional process of senescence and inflammaging, further research is needed.

Senotherapeutics are one of the approaches for removal of senescent cells (senolytics) or reducing inflammation (senomorphics) that are being investigated in vitro, ex vivo and in mice models in vivo [90,91,92,93]. However, we are yet to establish the long-term effects of these drugs, especially considering the long-term outcomes, potential off-target effects, and optimal timing for their administration. Similar is the case with our knowledge about the biologics and biomaterials that have been proposed to have anti-aging potential for BM-related cell populations. We are yet to conduct longitudinal studies ideally spanning subjects of a wide age range, to enable tracking of age-related changes before these changes turn to diseases.

**Figure 3:**
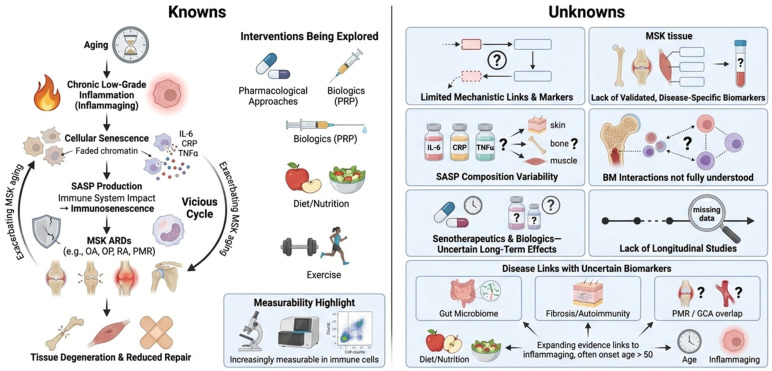
Summary of the knowns (**left side**) and unknowns (**right side**) of inflammaging markers (figure generated by AI platform FigureLabs).

Finally, there is increasing evidence of links between MSK and the gut microbiome and dysbiosis [94,95], fibrosis of skin in diseases like systemic sclerosis [96,97] and overlapping diseases like polymyalgia rheumatica (PMR) which is an MSK disease and giant cell arteritis (GCA) which is a vascular disease [98,99,100]. While several of these diseases are autoimmune, the common link among all of these diseases is advancing age, and expanding evidence suggests inflammaging as a causative factor due to the increased inflammation and the average onset age of patients in these diseases (over 50). Diving deeper into senescence and inflammaging will potentially unravel intricate links not just in MSK-related ARDs but also ARDs from other physiological systems. The knowns and the unknowns are briefly outlined below in Figure 3.

## 4. Challenges, Conclusions and Future Directions

While we are learning more and more about the field with every passing day, we are yet to determine succinct ways to define success in the field [101]. Heterogeneity in the aging trajectories of different organs, physiological systems and in us as human beings is what makes investigating the field challenging, yet profoundly interesting [102,103,104]. It is this variation that creates the debate between ‘chronological age’ and the ‘biological age’ of a person, and if the biological age can be lower than the chronological age, then it is a step towards healthy aging, disease-free life in the elderly and longevity. Next, the challenge of standardization and harmonization of biomarkers spanning various biological samples, geographical areas and across different research groups at a global scale is significant.

The information is fragmented and will need a methodical approach to identify consensus. Addressing the heterogeneity in aging trajectories, the consensus might need to be different for different organ systems, at least to begin with. The models used to investigate senescence and inflammaging are mainly in vitro, some ex vivo and animal-based in vivo models. Specifically, knockout mice models have provided critical information on genes and pathways that play a key role in MSK aging and associated ARDs like OP and osteosarcopenia (Table 2). While these models have evidently played a critical role in progressing further research, they have lacked translatability into application in human aging studies due to the incomplete biomimetic nature of these animal models. Thus, considerations must be made to use relevant three-dimensional (3D) models (organ-on-chip, organoids, scaffold based systems and spheroids), as the former models do not replicate the physiological condition in human MSK system [105].

The progressively expanding evidence base in the field underscores the pivotal roles of inflammaging and cellular senescence in driving the onset and progression of MSK diseases, despite the major gaps in deciphering their mechanistic interplay. While established hallmarks such as chronic low-grade inflammation, SASP-mediated tissue damage, and immune dysfunction provide a solid foundation for understanding MSK degeneration, the field still needs precise biomarkers to reliably capture disease stage, tissue specificity, and therapeutic responses. Most importantly, the fact that the most accepted inflammaging biomarkers are essentially inflammation markers cannot be ignored. More specific biomarkers indicating the age-related changes at the cellular level will play a critical role in identifying inflammaging and translating the knowledge towards potential therapies. Bridging these gaps will require bold, integrative, multi-omics approaches with enhanced translational models, and relevant clinical studies. Advancing biomarker discovery will not only deepen our understanding of MSK aging but also pave the way for targeted, preventive, and personalized interventions that can meaningfully improve long-term MSK health in the elderly.

Future research on biomarkers of inflammaging and cellular senescence in MSK health and diseases must focus on resolving the current gaps in mechanistic understanding, enhancing translational relevance, and targeting diagnostic precision. Translating aging biomarkers into diagnostics and therapeutics for inflammaging will need a structured and step-wise strategy. First and foremost, validated biomarkers (e.g., IL-6, CRP, senescence markers) will need to be prioritized with reproducible links to clinical outcomes like early diagnosis. Next, integrating multi-omics and functional measures (e.g., epigenetic clocks, gait speed) will play a critical role in improving sensitivity and disease relevance. Next, longitudinal cohorts and clinical trials must be utilized, to establish causality and predictive value, followed by the standardization of assays and thresholds to enable clinical adoption. Finally, biomarkers must be directly associated with targetable pathways, allowing development of gero-therapeutics such as anti-inflammatory or senolytic interventions, ensuring true translational impact.

Emerging multi-omics, timely utilization of artificial intelligence (AI), next-generation technologies, spatial profiling, and single-cell approaches offer promising opportunities to map senescence–immune interactions with higher resolution and to identify tissue-specific biomarker signatures [112,113,114]. Longitudinal human studies will be essential to validate these markers, establish aging biomarkers and pathways, and determine their predictive value for disease progression. In parallel, integrating biomarker discovery with interventional trials, spanning senolytics, senomorphics, SASP modulators, microbiome-targeted strategies, and lifestyle interventions, will help provide further clarity towards pathways and optimize the stage at which these interventions may be administered. Ultimately, these advances promise to enable earlier detection, personalized risk stratification, and targeted therapies that can transform the future of MSK disease management.

## Figures and Tables

**Table 1:** Examples of studies investigating different biomarkers in MSK aging.

Biomarker Category	Target Organ	Key Biomarkers Investigated	Reference
Biological	Muscle	IGF-1, myostatin, IL-6, irisin, IL-15	[29]
Biological	Muscle	Titin fragments (TTN), miR-451a	[30]
Biological	Joint	*FOXO3*, *SIRT3*, *MCL1*, *STAG1*	[31]
Biological	Joint	Inflammatory mediators, ncRNA, metabolites	[32]
Functional	Muscle	Grip strength, gait speed	[33]
Digital	Overall fitness	Gait speed, stride length, variability	[34]
Biological	Across tissues	p21, p16	[35]
Biological	Bone	*klotho*	[36]
Biological	Bone	*Sirtuins*	[37]

**Table 2:** Representative knockout studies investigating MSK aging.

Investigation	Knockout Model	Aging Focus or ARD	Key Results	Reference
Hepcidin1 KO osteoporosis	Global KO mice	Bone aging/osteoporosis	Iron overload increases oxidative stress, ↓ osteoblasts, ↑ osteoclasts	[106]
DDR1 KO bone aging	Global KO mice	Bone microarchitecture aging	Age-dependent defects in bone remodeling, impaired osteoclastogenesis	[107]
IL-21 KO osteoporosis	Global KO + OVX	Postmenopausal bone loss	Reduced RANKL signaling and osteoclastogenesis	[108]
Akt1/2 muscle KO osteosarcopenia	Muscle-specific KO	Muscle + bone aging	Loss of muscle mass, osteopenia, reduced lifespan via insulin/FoxO pathway	[109]
PRMT5 myogenic KO	Myod1-Cre KO	Muscle aging/regeneration	Impaired satellite cells, ↑ FoxO1, autophagy dysregulation	[110]
Asb2 muscle KO	Muscle-specific KO	Sarcopenia resistance	↑ muscle mass and strength, reduced protein degradation	[111]
Kl skeletal architecture in aging	*Klotho*-mutant	Chronological aging	*Kl*/*kl* produces an accelerated aging phenotype partially	[36]

↓: decreases/decreased, ↑: increases/increased.

## Data Availability

No new data were created or analyzed in this study.

## Abbreviations

The following abbreviations are used in this manuscript:

3D	Three-dimensional
AGE	Advanced glycation end-product
AI	Artificial intelligence
ARD	Age-related disease
BM	Bone marrow
CRP	C-reactive protein
CTX	C-terminal telopeptide
DDR	DNA damage response
GDF-15	Growth differentiation factor 15
IGF-1	Insulin-like growth factor 1
IL-1	Interleukin-1
IL-6	Interleukin-6
MSCs	Mesenchymal stromal/stem cells
MSK	Musculoskeletal
OA	Osteoarthritis
OP	Osteoporosis
PINP	Procollagen type I N-propeptide
PRP	Platelet-rich plasma
QOL	Quality of life
RA	Rheumatoid arthritis
ROS	Reactive oxygen species
SA-β-gal	Senescence-associated beta-galactosidase
SASP	Senescence-associated secretory phenotype
T2D	Type 2 Diabetes
TNFα	Tumor necrosis factor alpha
WHO	World Health Organization
